# Haplotypes traceability and genetic variability of the breeding population of pacu (*Piaractus mesopotamicus*) revealed by mitochondrial DNA

**DOI:** 10.1590/1678-4685-GMB-2020-0249

**Published:** 2021-03-12

**Authors:** Milena V. de Freitas, Raquel B. Ariede, Milene E. Hata, Vito A. Mastrochirico-Filho, Felipe Del Pazo, Gabriela V. Villanova, Fernando F. Mendonça, Fábio Porto-Foresti, Diogo T. Hashimoto

**Affiliations:** 1 Universidade Estadual Paulista "Júlio de Mesquita Filho" (UNESP), Centro de Aquicultura, Jaboticabal, SP, Brazil.; 2 Universidad Nacional de Rosario, Facultad de Ciencias Bioquímicas y Farmacéuticas - Ministerio de Ciencia, Tecnología e Innovación productiva de Santa Fe, Centro Científico y Tecnológico Acuario del Río Paraná, Rosario, Santa Fe, Argentina.; 3 Universidade Federal de São Paulo (UNIFESP), Instituto do Mar, Santos, SP, Brazil.; 4 Universidade Estadual Paulista "Júlio de Mesquita Filho" (UNESP), Faculdade de Ciências, Bauru, SP, Brazil.

**Keywords:** Control-region, genetic breeding, aquaculture

## Abstract

The main objective of this study was to estimate the genetic diversity levels and haplotype traceability in pacu *Piaractus mesopotamicus* from the breeding program located in Brazil by analyses of the mitochondrial DNA control region (mtDNA). Moreover, broodstocks from eight commercial fish farms were used for comparative evaluation, four from Brazil (Br1-Br4) and four from Argentina (Ar1-Ar4). The descriptive results revealed 47 polymorphic sites and 51 mutations, which evidenced 34 haplotypes. Ten haplotypes were shared among fish farms and 24 were exclusive. The nucleotide diversity (π) ranged from 0.00031 to 0.01462 and haplotype diversity (Hd) from 0.125 to 0.868. The analysis of molecular variance (AMOVA) indicated high structure present in the analyzed stocks (*F*_*ST*_ = 0.13356 and Ф_ST_ = 0.52707). The genetic diversity was high in most of the commercial broodstocks, especially those from Brazil. We observed seven haplotypes in the genetic breeding population, of which four were exclusive and three shared among the commercial fish farms. The genetic diversity was moderate (π = 0.00265 and Hd = 0.424) and considered appropriated for this breeding population of pacu. Our results provide support for the genetic diversity maintenance and mtDNA traceability of pacu commercial broodstocks.

## Introduction

The species *Piaractus mesopotamicus*, popularly known as pacu, is a Serrasalmidae fish of commercial importance distributed in the Paraná, Paraguay, and Uruguay River basins, mainly in the Pantanal plains (Petrere Jr, 1989). Pacu is one of the main farmed fish of the South American aquaculture, occupying the 6th position of production in Brazil, together with its hybrid patinga (obtained by crossing *P. mesopotamicus* and *Piaractus brachypomus*), with an estimated annual production of 13,276 tons (IBGE, 2015). In addition, pacu has a representative production in Argentina, where it is the main farmed fish produced (52.6% of the total production, 2,119 tons) (Schenone *et al*., 2011; Panné Huidobro, 2015); and in Asian countries (China, Myanmar, Thailand and Vietnam) (Flores Nava, 2007; Honglang, 2007; FAO, 2010; Lin *et al*., 2015; Valladão *et al*., 2018).

Currently, major breeding programs in aquaculture have been performed using a family scheme, which can lead to the selection of siblings (Gjedrem and Baranski, 2010). However, the formation of inbred families and the use of breeding units with low genetic variability result in loss of genetic potential and inbreeding risks (Melo *et al*., 2006; Charlesworth and Willis, 2009). In rainbow trout (*Oncorhynchus mykiss*), the different degrees of inbreeding depression significantly affected the percentage of hatching, fecundity and larval survival, as well as generating individuals with morphological deformities (Yosefian and Nejati, 2008). Although the characterization of genetic variability is a fundamental tool when initiating genetic breeding programs (Mastrochirico-Filho *et al*., 2019a), few studies have been developed in this scope. 

Recently, genetic diversity was assessed by nuclear markers SNPs (single-nucleotide polymorphisms) and microsatellites in different fish farms from Brazil for a pre-breeding program in *Piaractus mesopotamicus* (Mastrochirico-Filho *et al*., 2019a). These previous results have evidenced genetic variability and structure at low level in pacu farmed stocks, including fish profiles with risks of inbreeding. This information was exploited for the creation of the breeding population to select superior genotypes related to growth performance and disease resistance in pacu (Mastrochirico-Filho *et al*., 2019b; Freitas *et al*., 2020). Nowadays, supplementary studies are still required to follow up the level of genetic diversity of this breeding population (Mastrochirico-Filho *et al*., 2019b; Freitas *et al*., 2020) in pacu, particularly using the control region (CR) of the mitochondrial DNA (mtDNA) that has relatively lower mutation fixation rate than microsatellites and, therefore, is appropriated to capture genetic differences in older (non-contemporary) events and to assess traceability of fish products resulting from genetic improvement (Aquadro and Greenberg, 1983; Meyer, 1993; Brown *et al*., 1993).

The main objectives of this study were: 1) to assess the genetic diversity by mtDNA in the breeding population of pacu from Caunesp (Aquaculture Center of the São Paulo State University, Brazil); 2) to identify unique haplotypes in this breeding population for traceability of products resultant from genetic improvement by mtDNA; 3) to compare farmed stocks from different Brazilian and Argentinian commercial fish farms by mtDNA in order to detect non-contemporary genetic differences of founder stocks and to understand the haplotype diversity and distribution between these countries.

## Material and Methods

### Experimental populations

Pacu individuals *Piaractus mesopotamicus* from the breeding nucleus belonging to the Caunesp (Aquaculture Center, São Paulo State University, Jaboticabal, Brazil) were used for the genetic analysis. These breeders are resulting from the base population established with purposes of genetic selection for growth performance and disease resistance in pacu (Mastrochirico-Filho *et al*., 2019b; Freitas *et al*., 2020). In addition, samples from eight different commercial broodstocks from fish farms in Brazil and Argentina were also collected (number of individuals and fish farms are shown in Table 1). The commercial identity and localization of the fish farms were kept confidential. Animals were individually tagged with transponders (passive integrated transponder tags - pit-tags, model full-duplex FDX-B, 134.2 kHz) and kept alive for subsequent management. Fish farms were mostly set up between the 1980s and 1990s. There are no records of selective mating in the commercial broodstocks.

**Table 1 -:** Genetic parameters of mtDNA diversity in farmed individuals of *P. mesopotamicus*.

Origin	Fish Farms	N	S	h	Hd	π
Argentina	Ar1	16	1	2	0.233	0.00058
	Ar2	14	6	8	0.868	0.00640
	Ar3	16	1	2	0.125	0.00031
	Ar4	14	27	8	0.824	0.01462
Brazil	Br1	14	4	3	0.385	0.00198
	Br2	16	15	9	0.858	0.00917
	Br3	19	17	7	0.749	0.00671
	Br4	18	10	5	0.660	0.00894
	CAUNESP	41	13	7	0.424	0.00265
	All	168	47	34	0.656	0.00163

N= number of individuals, S= polymorphic sites, h= haplotypes, Hd= Haplotypic diversity, π= nucleotide diversity

Fin samples were collected from each fish under benzocaine solution (200 mg^L-1^) (Sigma, St. Louis, USA) anesthesia and all efforts were made to minimize suffering. Fin samples were stored in 95% ethanol at −20 °C.

### Amplification of the control region and sequencing

DNA extraction was carried out using the Wizard Genomic DNA Purification Kit (Promega). DNA integrity was evaluated on 1% agarose gel and its purity was assessed using a NanoDrop One spectrophotometer (Thermo Fisher, Madison, USA). The DNA concentration was quantified using the Qubit dsDNA BR Assay kit (Life Technologies, Oregon, USA) and measured in a Qubit 3.0 Fluorometer (Invitrogen, Kuala Lumpur, Malaysia).

Genetic diversity was assessed through the partial sequencing of the mitochondrial DNA control region (D-loop). The primers PM01 (5’GATCCCAGTACATTATATGTAT3’) and PM02 (5’CCTTGTTAATCATTACRCTGA3’) were designed using the software Geneious V.7.1.3, using the mitochondrial genome of *P. mesopotamicus* (NC_024940) as reference. PCR assays were performed at a final volume of 12 μl: 1X *Taq* polymerase buffer; 1.5 mM MgCl ^2^; 100 μM of each dNTP; 0.1 μM of each primer; 10-50 ng of genomic DNA and 0.5 U of Taq polymerase (Invitrogen). The following amplification cycle was used: denaturation 94 °C for 3 min, 35 cycles of denaturation at 94 °C for 1 min; annealing at 62 °C for 45, extension to 72 °C for 1 min, and a final extension at 72 °C for 10 min. PCR assays were performed on the ProFlex ™ PCR System (Life technologies) and the final product checked in 1.5% agarose gel.

PCR products were cleaned with EXO-SAP Kit (USB® ExoSAP-IT® PCR Product Cleanup) and then underwent PCR sequencing, using BigDYE, Terminator Cycle Sequencing Kit version 3, 1 (Applied Biosystems, Inc.). The sequencing was performed in the Center of Biological Resources and Genomic Biology (CREBIO), in UNESP - Jaboticabal, Brazil, using the ABI 3730 XL DNA Analyzer (Applied Biosystems).

### Statistical Analysis

The assembled sequences were analyzed manually using the program CLUSTALW (Thompson *et al*., 1994), included in the program Bioedit (Hall, 1999). The nucleotide compositions, sequence diversity, number of polymorphic areas, and haplotype diversity were calculated using the software DNAsp v.5 (Librado and Rozas, 2009). The Analysis of Molecular Variance (AMOVA) (Excoffier *et al*., 1992) was conducted to test the genetic heterogeneity between mtDNA haplotypes using ARLEQUIN version 3.01 (Excoffier *et al*., 2007), which uses Wright’s F-statistics (1951, 1965). Haplotype network was estimated by Software Network v 4.6.1.0. 

## Results

The results revealed 47 polymorphic sites from approximately 400 sequenced base pairs (bp), characterizing 34 haplotypes (GenBank accession numbers MW287387 - MW287554). Among the haplotypes, 10 were shared among broodstocks, of which two (H1 and H2) were shared between samples from Brazilian and Argentinian fish farms. In addition, 24 unique haplotypes were detected, of which 11 were in Argentina, 9 were in Brazil, and 4 were exclusive from the base population of the breeding nucleus (Table 2). The most representative haplotype was H1, which was distributed in all fish farms. The percentage of bases among the haplotypes corresponded to 28.4% Adenine (A), 33.4% Thymine (T), 22.1% Cytosine (C) and 16.1% Guanine (G).

**Table 2 -:** mtDNA haplotypes distribution among fish farms (Ar and Br) and the breeding nucleus (CAUNESP).

h	Ar1	Ar2	Ar3	Ar4	Br1	Br2	Br3	Br4	CAUNESP	All	%
h1	14	4	6	15	8	11	6	1	12	77	51.6
h2	2	1		1		2	2			8	5.3
h3		4								4	2.6
h4		1								1	0.6
h5		1								1	0.6
h6		1								1	0.6
h7		1								1	0.6
h8		1								1	0.6
h9						1				1	0.6
h10					6		1	10	4	21	14.0
h11							1			1	0.6
h12							2			2	1.3
h13							1			1	0.6
h14							1			1	0.6
h15					1		1			2	1.3
h16							1			1	0.6
h17					1			4		5	3.3
h18					1					1	0.6
h19					1					1	0.6
h20					1					1	0.6
h21								2		2	1.3
h22								1		1	0.6
h23			2							2	1.3
h24			1							1	0.6
h25			1							1	0.6
h26			1							1	0.6
h27			1							1	0.6
h28			1							1	0.6
h29			1							1	0.6
h30									1	1	0.6
h31									2	2	1.3
h32									1	1	0.6
h33									1	1	0.6
h34									1	1	0.6

The nucleotide (π) and haplotypic (Hd) diversity demonstrated moderate values in the base population of the breeding nucleus (π = 0.00265 and Hd = 0.424) (Table 1) and indicated two distinct patterns of genetic variability in the commercial fish farms, with stocks with high and low genetic diversity. The highest haplotype diversity was found in Ar2 (Hd = 0.868), and the lowest diversity in Ar3 (Hd = 0.125). The nucleotide diversity was higher in Ar4 (π = 0.01451) and lower in Ar3 (π = 0.00031). In general, the genetic diversity was high (Global/Total Hd = 0.656 and π = 0.00163). The genetic variability was moderate (π = 0.00265 and Hd = 0.424) for this breeding population of pacu.

For the calculation of molecular variance (AMOVA) (Table 3), the populations were clustered according to the origin of the fish farm (Argentina and Brazil). The highest genetic variation was found within the populations. The *F*_*ST*_ index indicated a high genetic structure among the populations (*F*_*ST*_ = 0.13356), similarly to the Ф_ST_ index (0.52707, p <0.05). The pairwise *F*_*ST*_ matrix also revealed high genetic structure between the broodstocks (Table 4), particularly comparing the broodstocks from Argentina.

**Figure 1 -:**
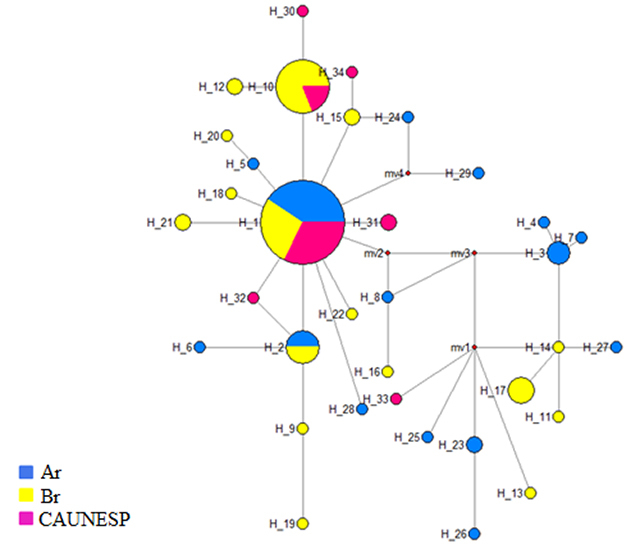
Network of haplotypes in relation to haplotype flow among fish farms. The size of the circle is proportional to the haplotype frequency. Ar is fish farms from Argentina, Br is fish farms from Brazil, and CAUNESP from the breeding nucleus.

The haplotype network (Figure 1) revealed the H1 haplotype as ancestral, and the consequent establishment of the other haplotypes. There was no evident distribution pattern of the haplotypes between the fish farms from Brazil and Argentina, and only the H1 and H2 haplotypes were present in the fish farms from both countries.

**Table 3 -:** Analysis of molecular variance (AMOVA).

AMOVA			
	Variance Components	percentage of variation (%)	*F_*ST*_
Among groups	-0.02591	-2.16	0.13356
Among populations within groups	0.18649	15.51	*ɸ_ST_
Within populations	1.04173	86.64	0.52707

*Significant p values p<0.05

**Table 4 -:** Differentiation index (*F*_*ST*_) between pairs of broodstocks of fish farms from Argentina, Brazil, and the breeding nucleus (CAUNESP) of *Piaractus mesopotamicus*.

	Ar1	Ar2	Ar3	Ar4	Br1	Br2	Br3	Br4	CAUNESP
Ar1		**0.31846**	-0.04242	**0.15974**	-0.02008	**0.08905**	**0.10289**	**0.30470**	0.02456
Ar2			**0.33508**	**0.08766**	**0.26022**	**0.06663**	**0.19733**	**0.22388**	**0.31749**
Ar3				**0.16055**	**0.02088**	**0.09789**	**0.10117**	**0.30803**	0.01479
Ar4					0.13960	0.01856	**0.10984**	**0.11313**	**0.20089**
Br1						0.05729	**0.08362**	**0.27399**	0.05071
Br2							0.01570	0.05811	**0.08390**
Br3								0.06433	0.03517
Br4									**0.25143**
CAUNESP									

Pairwise F_st_ numbers are above de diagonal line. and the significant of the p-values are in bold.

## Discussion

Inbreeding depression is one of the factors that most affects individuals due to the classical (phenotype-based) selection method in the breeding nucleus. The main genetic problems arising from the inappropriate use of fish in breeding programs can be reduced and even avoided using the genetic profile of the individuals and proper genetic management practices, similarly as it was performed to compose the base population of the breeding nucleus (CAUNESP) in pacu (Mastrochirico-Filho *et al*., 2019b; Freitas *et al*., 2020). This was supported by the analysis of the present study, which revealed a moderate pattern of genetic diversity in this breeding population. Moreover, the mtDNA results can be also applied to maximize the genetic variability in the subsequent generations of the selection process in pacu, considering the composition of different haplotypes during the selection steps. The data herein obtained also provides exclusive markers for the genetic traceability of the products resulting from the genetic improvement process, especially the unique haplotypes detected in this initial base population, which will later allow identifying their progenies as products from selection process. However, studies approaching additional samples and fish farms will be still necessary to corroborate the practical and reliable applicability of these haplotypes for traceability analysis.

Our results indicated high values of genetic variability in most of the commercial broodstocks of farmed pacu, similarly to the genetic pattern of wild populations also using mtDNA (Iervolino *et al*., 2010), which represents the potential use of these stocks in genetic selection programs. However, three broodstocks registered low variability rates (Ar1, Ar3 and Br1), similar to those obtained in others farmed broodstocks analyzed by SNP and microsatellite markers (Mastrochirico-Filho *et al*., 2019a), which detected low values of allele number and heterozygosity. Low genetic diversity may represent populations with genetic drift events due to low effective population sizes and recent bottleneck effects/founder events (Mastrochirico-Filho *et al*., 2019a); therefore, the fish farms Ar1, Ar3 and Br1 ought to introduce/replace new breeders with different genetic background, particularly to avoid inbreeding depression that compromises the foundation of hatchery stocks when initiating breeding programs (Duncan *et al*., 2013; Naish *et al*., 2013).

The data of the genetic structure indicated the existence of low gene flow among the different stocks of fish farms in Brazil and Argentina. This pattern is expected for the present populations, as the broodstocks are geographically isolated and producers frequently do not exchange breeders among the fish farms, similar what was already detected in a closely related species *Piaractus brachypomus* by microsatellites (Jorge *et al*., 2018). In contrast, Mastrochirico-Filho *et al*. (2019a) demonstrated low differentiation between different farmed stocks from Brazil, which could be attributed to stock foundation based on breeders sharing among fish farms, and/or stock foundation in the fish farms based on the capture of wild breeders, which are characterized as belonging to a panmictic unit due to the lack of genetic structure in natural populations (Iervolino *et al*., 2010), particularly because pacu have high gene flow capacity due to their migratory behavior in the wild.

The genetic results of this study generated an improved knowledge of the mitochondrial profile of pacu commercial broodstocks in Brazil and Argentina, which provides a framework for the development of management programs and genetic improvement of this species in aquaculture. In addition, in terms of the genetic composition of the breeding program, the base population showed moderated levels of genetic variability compatible with the wild stocks (high haplotypic and nucleotide diversity), which would reduce the problems related from narrowing the genetic base or loss of genetic potential over the various generations of selection.
